# Antioxidant Potential of *Mangifera foetida* Bark, *Mangifera foetida* Leaves, and *Cinnamomum burmanii* Leaves Extract in Mitigating Nanoplastic‐Induced Toxicity and Disruption of Glycolipid Metabolism

**DOI:** 10.1155/adpp/7285762

**Published:** 2025-09-16

**Authors:** Manikya Pramudya, Raden Joko Kuncoroningrat Susilo, Windy Seftiarini, Firli Rahmah Primula Dewi, Farah Annisa Nurbani, Aulia Umi Rohmatika, Aunurohim, Vuanghao Lim, Alfiah Hayati

**Affiliations:** ^1^ Department of Biology, Faculty of Science and Technology, Airlangga University, Campus C Mulyorejo, Surabaya, 60115, Indonesia, unair.ac.id; ^2^ Department of Engineering, Faculty of Advanced Technology and Multidiscipline, Airlangga University, Campus C Mulyorejo, Surabaya, 60115, Indonesia, unair.ac.id; ^3^ Master’s Program in Biotechnology, Graduate School, Gadjah Mada University, Teknika Utara Street Kocoran Caturtunggal Depok, Sleman, 5528, Daerah Istimewa Yogyakarta, Indonesia, ugm.ac.id; ^4^ Department of Biology Education, Faculty of Teacher Training and Education, Sriwijaya University, Inderalaya, 30662, South Sumatera, Indonesia, unsri.ac.id; ^5^ Faculty of Medicine, UPN “Veteran” East Java, Surabaya, Indonesia; ^6^ Department of Biology, Faculty of Science and Data Analytics, Sepuluh Nopember Institute of Technology, Campus ITS Sukolilo, Surabaya, 60111, Indonesia, its.ac.id; ^7^ Department of Toxicology, Advanced Medical and Dental Institute, Universiti Sains Malaysia, Bertam 13200 Kepala Batas, Pulau Pinang, Malaysia, usm.my

**Keywords:** antioxidants, health, nanoplastic, oxidative stress, plant extract

## Abstract

Pollution from plastic waste has become an urgent issue, requiring solutions to prevent and mitigate diseases caused by plastic waste, particularly those involving nanoplastics (NP). This study specifically focused on investigating the exogenous antioxidant activity of three plant extracts: *Mangifera foetida* bark (MFB), *Mangifera foetida* leaves (MFL), and *Cinnamomum burmanii* leaves (CBL), to enhance the body’s defense system and reduce the risk of Type II diabetes. Twenty‐five rats (*Rattus norvegicus*) were randomly assigned to five groups: normal control, negative control, and three treatments that received different plant extracts (200 mg/kg of MFB, MFL, and CBL, respectively) after being exposed to 10 μL/kg NP for 30 days. According to confocal microscopy analysis, NPs were observed entering cells and localizing in the nucleolus more than in the cytoplasmic hepatocyte. This study found that the administration of the plant extract could reduce the level of the proapoptotic enzyme not through the intrinsic pathway but via the extrinsic pathway. Administration of MFB, MFL, and CBL could reduce Caspase‐3 significantly (1.07 ± 0.05, 1.03 ± 0.08, 1.05 ± 0.10 ng/L, respectively). This effect is mediated by the upregulation of genes related to glycolipid metabolism, including AKT2, GLUT2, PI3K, FAS, PEPCK, and PK. Administration of MFL significantly upregulated the expression levels of AK2, GLUT2, PI3K, and PK genes compared to the negative control. Administration of CBL extract enhanced the percentage of normal hepatocytes and the diameter of the central vein and decreased the percentage of necrosis, swelling, and the number of Kupffer cells. All treatment groups showed a slight decrease in the level of SGOT and SGPT. Thus, plant extracts could be effective materials exhibiting exogenous antioxidant activity against NP, directly inhibiting proapoptotic signals and regulating glycolipid metabolism. These extracts could be further developed as a preventive or therapeutic strategy to address NP exposure in environmental and clinical settings.

## 1. Introduction

Plastic is a widely used polymer material due to its lightweight, practicality, strength, affordability, and flexibility [1]. However, inadequate waste management systems can lead to serious health problems. Plastic waste incineration [2], lack of proper processing of contaminated water sources before consumption [3–5], and friction between car tires and asphalt can contribute to releasing plastic particles [6]. In addition, commercial table salt from seawater evaporation [7, 8], cosmetics, and personal care products such as toothpaste, face cream, and lipstick often contain nanoplastics (NP) such as polyethylene or polyethylene terephthalate (PET) [9]. As a result, NP particles can easily enter the body through inhalation, ingestion, or skin surface [10–12].

NP are resistant to natural degradation due to their strong durability [9]. However, environmental stress factors such as ultraviolet radiation and high temperatures can degrade plastics into smaller particles [13]. These degraded plastics can be categorized into microplastics (1 μm–5 mm) and NP (less than 1000 nm or 1 μm) [14]. When plastic particles enter water systems and water processing facilities cannot filter out NP due to their small size, they cause pollution that contaminates aquatic organisms consumed by humans, such as fish, shrimp, and squid, leading to serious health problems [15–18]. NP exposure in the body can cause oxidative stress, affecting immune responses and reproductive health [19]. The toxicity of NP is determined by their characteristics and sources of production [20], and their small size can trigger cellular‐level toxicity, raising concerns [21]. NP particles can enter cells through endocytosis, disrupting the function of endocytic pathways and endangering endosome membranes [22, 23]. NP particles are smaller than cell membrane pores. They can penetrate the intestinal barrier, disrupt cell surface receptor signaling, and alter gene expression in the nucleus. High concentrations of NP can disrupt membrane permeability, allowing them to penetrate cell membranes into the cytosol.

Studies have shown that NP accumulation can lead to the production of reactive oxygen species (ROS) [24–26]. The overproduction of ROS leads to an imbalance between ROS and the cellular antioxidants [27]. High levels of ROS cause cell damage or apoptosis through FAS‐related signaling pathways [28]. ROS also increases p53 activation, which releases proapoptotic proteins (Bax family) and antiapoptotic proteins (Bcl‐2), leading to mitochondrial cytochrome release and activation of Caspase‐9 and Caspase‐3. Caspase‐9 and Caspase‐3 are the primary mediators of apoptotic cell death [29]. In addition, elevated levels of ROS increase the expression of p53, gadd45ba, and Caspase‐3, leading to DNA damage and inducing an apoptosis response that is dependent on both dose and duration. ROS‐mediated cell damage, indicated by lipid peroxidation (LPO), involves sphingomyelinase activation and ceramide release, ultimately causing cell death [30]. ROS formation can also trigger the transcription of cytoprotective genes by releasing endogenous antioxidants (CAT/SOD/GST/GPX), which inhibit oxidative stress and prevent cell damage [31].

NP exposure impacts glycolipid metabolism, a vital component of cell membranes essential for structural stability and intercellular signaling. Glycolipids, composed of lipid molecules bound to carbohydrates, play critical roles in molecular transport, immune responses, and cell differentiation. NP disrupt this metabolism through enzyme dysregulation, affecting the expression and activity of enzymes involved in glycolipid synthesis or degradation, such as gangliosides and glucosylceramides. In addition, NP trigger LPO, damaging glycolipids as integral plasma membrane components, and impair intercellular communication, particularly in the nervous system, where lipid–carbohydrate interactions are crucial for synaptic function [32]. Disruption of glycolipid metabolism due to NP exposure can lead to various diseases, including systemic metabolic disorders, where imbalances in glycolipid metabolism may cause insulin resistance and obesity, which are the major risk factors for Type 2 diabetes. In addition, neurodegenerative disorders may arise, as glycolipid damage contributes to synaptic dysfunction associated with conditions such as Alzheimer’s or Parkinson’s disease [33].

High ROS levels in cell plasma can disrupt the phosphatidylinositol 3‐kinase (PI3K)/phosphoprotein kinase B (p‐AKT)/glucose transporter 4 (GLUT4) pathway in glucose metabolism, leading to insulin resistance and increased serum glucose and lipoprotein levels [34, 35]. Insulin, a peptide hormone responsible for energy storage and metabolism, stimulates the release of glucose from pancreatic β cells. The presence of ROS inhibits the phosphorylation activation of the insulin receptor substrate (IRS), which in turn inhibits the activities of the PI3K/AKT signaling pathway. This blockage affects transcription factors such as forkhead box O1 (FOXO1), impacting glucose supply, cell metabolism, and growth, especially in pancreatic β‐cell functions [36]. In the liver, the transcription factor FOXO1, regulated by insulin under physiological conditions, plays a dominant role in stimulating gluconeogenesis. Disturbance in FOXO1 transcription causes insulin signaling to be blocked, driving gluconeogenesis and leading to excessive glucose production in Type 2 diabetes [37, 38]. Hyperglycemia and hyperlipidemia can trigger oxidative stress and glycolipid metabolism disorders, causing organelle dysfunction and contributing to the pathogenesis of Type 2 diabetes, especially in vascular complications, leading to endothelial cell damage before insulin resistance [39].

Increased ROS can be neutralized by antioxidant compounds, which release one of their electrons to stabilize unstable molecules due to electron deficiency [40]. One natural material with strong antioxidant potential is *Cinnamomum burmannii*, which contains active flavonoids and phenolic compounds. Phenolic compounds and flavonoids are antioxidants with potent exogenous antioxidant properties [41–43]. *Mangifera foetida,* one of the plants belonging to the family of Anacardiaceae also contains flavonoids predominantly as secondary metabolites. Furthermore, *M. foetida* contain 2.56% more mangiferin compared to other mango varieties [44]. Mangiferin (2‐C‐β‐D‐glucopyranosyl‐1,3,6,7‐tetrahydroxyxanthone) is a polyphenolic compound known for its potent antioxidant properties and numerous pharmacological activities. Compounds such as cinnamaldehyde in *C. burmannii* can neutralize ROS, repair cellular damage, and restore the function of the body’s antioxidant enzymes [45]. Toxicity tests on rodents have shown that mangiferin compounds do not exhibit toxic properties in both acute and subchronic conditions [46]. Although the body has developed defense systems to eliminate ROS through endogenous enzymes, an imbalance between free radicals and antioxidants can cause protein and LPO damage [47]. However, there is still limited information about the impact of *C. burmanii* and *M. foetida* on the physiology of rats exposed to NP. While NP toxicity is an emerging environmental and health concern, the application of these plant extracts as natural antioxidants to counteract oxidative stress and cellular damage has not been widely explored. This approach not only provides new insights into the protective potential of these extracts but also addresses a significant gap in the research on sustainable strategies to combat NP‐related health risks.

Emerging evidence suggests that chronic exposure to NP may play a contributory role in the development of metabolic disorders, including Type 2 diabetes mellitus. NP‐induced oxidative stress can interfere with insulin receptor signaling and trigger systemic inflammation, both of which are key mechanisms underlying insulin resistance, a central feature of Type 2 diabetes mellitus [48, 49]. Prolonged oxidative imbalance caused by NP exposure elevates proinflammatory cytokines such as TNF‐α and IL‐6, which impair IRS phosphorylation and inhibit downstream signaling pathways such as PI3K/AKT [50]. In addition, NP accumulation in metabolic organs such as the liver and pancreas may alter lipid and glucose metabolism, leading to hyperglycemia and dyslipidemia [51]. These findings support the hypothesis that NP, as an environmental pollutant, may serve as a novel risk factor for diabetes, especially when combined with predisposing factors such as poor diet, sedentary lifestyle, or genetic susceptibility.

In this research, we specifically investigated the exogenous antioxidant activity of three plant extracts (*M. foetida* bark [MFB], *M. foetida* leaves [MFL], and *C. burmanii* leaves [CBL]) to neutralize ROS from NP, enhance the body’s defense system and reduce the risk of Type 2 diabetes through cell damage and apoptosis pathways. This research is crucial to minimizing the health risks posed by NP.

## 2. Materials and Methods

### 2.1. Materials and Chemicals

Polystyrene NP (100 nm in size) were obtained from Sigma‐Aldrich Solution (Merck Millipore, Darmstadt, Germany). Caspase‐3 (catalog no. E1648.Ra), Caspase‐9 (catalog no. E0627Ra), and Bax (catalog no. E1869Ra) kits were obtained from BT‐Lab ELISA kit (Bioassay Technology Laboratory, Shanghai, China). RNA isolation kit was obtained from Geneaid RT050/RTD050 Total Mini Kit (Geneaid, New Taipei City, Taiwan). RT‐PCR and qPCR kits were obtained from Toyobo Thunderbird Next SYBR qPCR Mix 2103 (Toyobo Inc., Osaka, Japan). All chemical reagents used in this research were of analytical reagent grade.

### 2.2. The Animals

Male Wistar rats (200–220 g) were procured from the Faculty of Pharmacy, Universitas Airlangga, Surabaya, Indonesia. All experimental protocols were authorized by the Faculty of Dental Medicine (Ethical Clearance Commission), Universitas Airlangga, Indonesia (ethical clearance number: 1157/HRECC.FODM/X2023). These rats were placed under standardized controlled conditions (room temperature and balanced light‐dark cycle). They were given free access to both food and water for the entirety of the experimental period.

### 2.3. Preparation of Plant Extract

MFB, MFL, and CBL were obtained from the Purwodadi Botanical Garden, Pasuruan, Indonesia. All plants were identified by expert botanist Dr. Junairiah from the Department of Biology, Universitas Airlangga, with specimen vouchers (MFB and MFL: 01/05.MF/2023; CBL: 01/12.CB/2024). MFB, MFL, and CBL were left to air‐dry at room temperature until they reached a consistent weight. Once dried, they were ground into powder separately. Each of the dried materials (500 g) was soaked in 1500 mL of 96% ethanol for 48 h. The resultant mixture was subsequently subjected to evaporation (60°C–70°C water bath temperature). Afterward, the extract underwent lyophilization, following the method described by Ugwah‐Oguejiofor et al. [52]. Before being administered to the experimental animals, the extract was reconstituted in distilled water daily.

### 2.4. Study Design and Experimental Procedure

**Figure 1:**
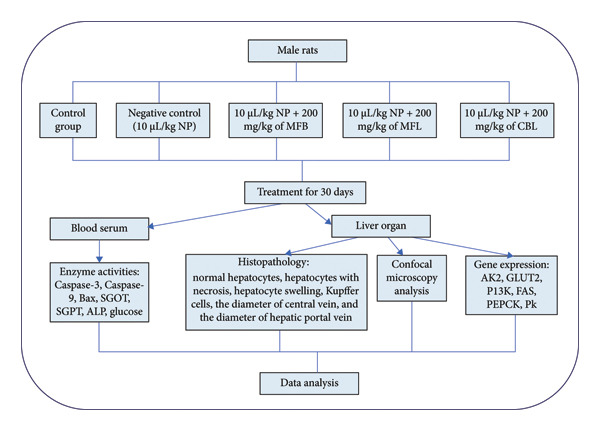
Experimental design.

The acclimatization period lasted for 2 weeks. Rats were randomly assigned to five treatment groups, including a control group (only sterile water, without NP and plant extracts), a negative control group (10 μL/kg NP), and three treatment groups that received different plant extracts (200 mg/kg of MFB, MFL, and CBL, respectively) after being exposed to 10 μL/kg NP. The experimental design is shown in Figure 1. The selection of the extract dose was based on a previous study [53, 54]. Both the NP and plant extracts were administered by gavage at a dose of 0.3 mL daily, with NP given in the morning (08:00) and plant extracts in the afternoon (16:00), with an 8‐h interval between administrations. The dose of NP was selected based on a prior study [55]. After the treatment period of 30 days, the animals were sacrificed. According to Cicero et al. (2018) [56], with modifications, before being sacrificed, the animals were first anesthetized intramuscularly using ketamine and xylazine. After the rats were anesthetized, blood was collected using a 23G injection needle. The collected blood was placed into a blood collection tube without EDTA. The blood was left at room temperature for 2 h, and then centrifuged at 3000 rpm for 10 min to separate it into two phases, and the supernatant (serum) was extracted. All experimental protocols were authorized by the Faculty of Dental Medicine (Ethical Clearance Commission), Universitas Airlangga, Indonesia (ethical clearance number: 1157/HRECC.FODM/X2023).

### 2.5. Confocal Microscopy Analysis

Rat liver was collected and washed with phosphate buffer saline to remove any residual blood. The liver was then fixed in 10% neutral buffer formalin (NBF) for 24 h. Subsequently, the tissue was processed for embedding in paraffin and sectioned with a microtome to a thickness of approximately 4–5 μm. The tissue sections were placed on gelatin‐coated glass slides to ensure the tissue remained adhered [57]. For observing NP distribution and localization, hepatocytes were stained with 1 μM 4′,6‐diamidino‐2‐phenylindole (DAPI) (Sigma) and 1 μM Nile red (Sigma). Using fluorescence techniques or confocal microscopy, Nile red produces a red coloration. Meanwhile, DAPI, a fluorescent dye, was chosen to detect NP interactions with biomolecules such as proteins and to visualize the distribution of NP within cells or tissues [58]. For staining, 100 μL dye solutions (prepared in DMSO) were mixed with the cell suspension (9.9 mL) and kept at room temperature for 30 min in the dark. The cells were then washed thrice before acquiring images. The images were acquired using a confocal laser‐scanning microscope (Olympus). The 3D multichannel image processing was performed using Thermo Scientific HCS Studio 2.0 Cells Analysis Software.

### 2.6. Measurement of Caspase‐3, Caspase‐9, and Bax Levels

The levels of Caspase‐3, Caspase‐9, and Bax were measured to assess proapoptotic activity. Serum was collected after centrifugation of whole blood (3000 rpm, 10 min). The levels of Caspase‐3, Caspase‐9, and Bax were measured by the ELISA method according to the kit’s instructions [59]. 40 μL sample and 10 μL antibody were added to the sample wells. After administration of 50 μL streptavidin–HRP, wells were incubated for 60 min at 37°C. Washing was performed 5 times. 50 μL of Substrate solution A (tetrametylbenzidine) and 50 μL of Substrate solution B (H_2_O_2_) were added to all wells. After incubation for 10 mins at 37°C in the dark, 50 μL stop solution was added. Optical density (OD) values were read at 450 nm using a microplate reader. The levels of Caspase‐3, Caspase‐9, and Bax were determined using a standard curve. A standard curve was generated for each target protein by plotting the OD values against known concentrations of the corresponding standard. The standard concentration ranges were 0.375–6 ng/L for caspase 3, 50–800 ng/L for caspase 9, and 250–4000 ng/L for Bax. The curve was constructed using a regression model, and sample concentrations were interpolated from this curve.

### 2.7. RT‐PCR and qPCR

#### 2.7.1. Preparation of Rat Hepatic Organ

Hepatic organs were collected from rats and were taken from each treatment group. Liver was taken as much as 25 mg and then stored at −20°C.

#### 2.7.2. RNA Isolation and RT‐PCR

Liver RNA isolation was performed with Geneaid RT050/RTD050 Mini Total RNA kit (Geneaid Biotech Ltd., Taiwan) according to Pick et al. (2012) [60]. The result of RNA isolation was then converted into cDNA through RT‐PCR using ReverTra Ace^TM^ qPCR RT Master Mix (Toyobo Co., Ltd., Japan). 10 μL of the reagent solution, consisting of 5x RT Master Mix 2 μL, isolated RNA, and nuclease‐free water, was inserted into the RT‐PCR tool with incubation settings at 37°C for 15 min, 50°C incubation for 10 min, and 98°C for 5 min. The RT‐PCR results were stored at −20°C for further analysis.

#### 2.7.3. Real‐Time PCR (qPCR)

We observed glycolipid metabolism–related genes such as AKT2, GLUT2, PI3K, FAS, PEPCK, and PK. Primer designs for glycolipid metabolism–related genes are presented in Table 1. The qPCR reaction was performed using THUNDERBIRD^TM^ Next SYBR qPCR Mix (Toyobo, Japan) according to Dewi et al. [61]. Real‐time PCR cocktail consisted of 5 μL ddH20, 10 μL SYBR qPCR Mix, 1 μL primer forward, 1 μL primer reverse, and 3 μL DNA solution. The PCR reactions were performed in triplicate with cycles of 95°C for 10 min, then followed by 40 cycles of denaturation at 95°C for 10 s, and annealing/extension at 57.5°C for 5 s. The threshold was determined using automatic settings from MyGo Pro software (V3.5.21). The calculation of mRNA relative expression values was obtained through MyGo Pro (V3.5.21) software and Microsoft Excel.

**Table 1:** Primer sequence.

Gene	Sequences	
	Forward	Reverse
AKT2	5′‐GGA GCT CTG TTA GCA CCG TT‐3′	5′‐AGT GGA AAT CCA GTT CCG AGC‐3′
GLUT2	5′‐CCA GCA CAT ACG ACA CCA GAC G‐3′	5′‐CCA ACA TGG CTT TGA TCC TTC C‐3′
P13K	5′‐ACA TCG ACC TAC ACT TGG GG‐3′	5′‐TCC CCT CTC CCC AGT AGT TT‐3′
FAS	5′‐CAG GAA CAA CTC ATC CGT TCT CT‐3′	5′‐GGA CCG AGT AAT GCC GTT CA‐3′
PEPCK	5′‐GTC CCC CTT GTC TAC GAA GC‐3′	5′‐TGC ATG ATG ACC TTG CCC TTA‐3′
PK	5′‐CGT GGA CGA TGG GCT CAT CT‐3′	5′‐AGG TTC ACG CCC TTC TTG CT‐3′

### 2.8. Histopathological Analysis

Histological processing involved fixing rat liver tissues in 10% NBF to preserve the tissues. The tissue was processed manually using 70%, 80%, 90%, and 100% ethanol. The processed tissues were then embedded in a paraffin block and sectioned to a thickness of 5 μm using a rotary microtome. Tissue sections were stained using the hematoxylin and eosin (HE). Each section was scrutinized under a light microscope (Olympus CZ22 Binocular). Several random fields of view were selected, and the number of each type of cell within the graticule was counted. The percentage of each cell type relative to the total cell count was calculated as follows: (number of specific cells/total number of cells) × 100%. The percentage of normal hepatocytes, hepatocytes with necrosis, hepatocyte swelling, the number of Kupffer cells, the diameter of the central vein, and the diameter of the hepatic portal vein were evaluated. Normal hepatocytes have eosinophilic cytoplasm, a centrally located round nucleus, organized chromatin, and a clearly visible nucleolus. Cells with edema display enlarged and paler cytoplasm due to fluid accumulation (hydropic degeneration). Necrotic cells exhibit nuclei showing pyknosis (shrinkage), karyorrhexis (fragmentation), or karyolysis (disappearance of the nucleus). Their cytoplasm may appear eosinophilic. Kupffer cells are specialized macrophages located within the liver sinusoids.

### 2.9. Measurement of Serum Glutamic Oxaloacetic Transaminase (SGOT), Serum Glutamate Pyruvate Transaminase (SGPT), Alkaline Phosphatase (ALP), and Glucose Levels

To evaluate liver function impairment, the levels of SGOT, SGPT, as well as ALP and glucose in rat serum were examined. For serum samples, whole blood was collected in serum separation tubes, allowed to stand for 30 min, and then serum was separated by centrifugation (5–10 min, 3000 rpm). Total serum SGOT, SGPT, ALP, and glucose were measured on the Horiba Pentra C200 autoanalyzer (Clinical Chemistry Analyzer, France).

### 2.10. Data Analysis

NP distribution and localization and gene expression of AKT2, GLUT2, PI3K, FAS, PEPCK, and PK were analyzed descriptively by comparing samples. One‐way ANOVA was used to analyze levels of Caspase‐3, Caspase‐9, BAX, SGOT, SGPT, ALP, glucose, and histology parameters (probability level of 0.05%). All data obtained were processed using SPSS Software V.24 (IBM Corp, New York, USA).

## 3. Results

### 3.1. Localization of NP in Cells

**Figure 2:**
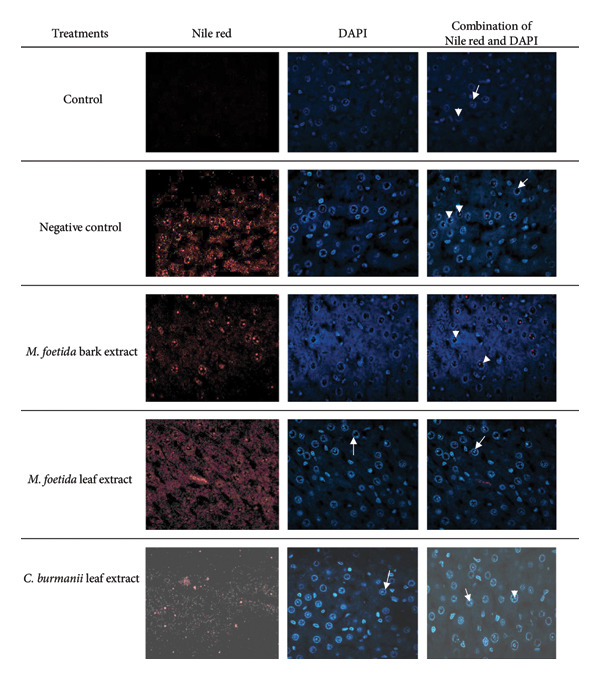
The presence of NP particles in cells with Nile red and DAPI staining. Red color = nanoplastics, white arrow = nucleus, and white arrow head = nucleolus. Magnification 100x. The control group: the nucleus and nucleolus of hepatocytes are visible with DAPI staining and the Nile red‐DAPI mix; negative control: many NP are observed with Nile red and Nile red‐DAPI mix but not with DAPI staining. NP is visible in most nucleoli of hepatocytes. The number of NP was found to be lower in the treatment with MFB, MFL, and CBL compared to the negative control.

According to Figure 2, the presence of NP was shown in cells stained with Nile red and DAPI. The presence of NP was shown in red color. In the control group, no NP was visualized. In the negative control group, many NP were visualized inside the cells, specifically in the nucleolus. Meanwhile, in the treatment groups with different types of plant extracts, NP were observed less frequently compared to the negative control.

### 3.2. Plant Extract Lower Proapoptotic Enzyme Level

Exposure to NP in their habitat leads to high proapoptotic enzyme levels, including Caspase‐3, Caspase‐9, and Bax (Table 2). The negative control showed a significant increase in Caspase‐3, Caspase‐9, and Bax levels (*p* < 0.01) compared to the control group. Administration of MFB and MFL significantly lowered Caspase‐3 levels compared to the negative control group (*p* < 0.05). The Caspase‐3 levels for MFB and MFL were 1.07 ± 0.05 and 1.03 ± 0.08 ng/L, respectively. The levels of Caspase‐9 and Bax also show significant differences after the administration of the plant extract compared to the negative control (*p* < 0.05).

**Table 2:** Proapoptotic enzyme level in rats following NP exposure and plant extract administration.

Groups	Proapoptotic enzyme (ng/L)		
	Caspase‐3	Caspase‐9	Bax
Control	0.87 ± 0.15	73.32 ± 9.46	349.80 ± 1.87
Negative control	1.38 ± 0.10^∗∗^	97.63 ± 0.84^∗^	471.99 ± 5.15^∗^
*M. foetida* bark extract	1.07 ± 0.05^∗^	103.62 ± 8.13^∗^	455.58 ± 69.89^∗^
*M. foetida* leaf extract	1.03 ± 0.08^∗^	99.69 ± 1.53^∗^	485.63 ± 59.19^∗^
*C. burmanii* leaf extract	1.05 ± 0.10^∗^	108.49 ± 5.36^∗∗^	526.23 ± 17.53^∗^

*Note:* Values are represented as mean ± SD (*n* = 5).

^∗^ *p* < 0.05.

^∗∗^ *p* < 0.01.

### 3.3. Plant Extract Increases the Expression of Glycolipid Metabolism–Related Gene

This study analyzed the expression of genes related to glycolipid metabolism. According to Figure 3, the negative control had the lowest percentage of AKT2, GLUT2, PI3K, FAS, PEPCK, and PK gene expressions compared to other groups. Our data revealed that MFL treatment significantly upregulated the expression of key genes involved in glycolipid metabolism compared to the negative control group. Specifically, MFL treatment significantly upregulated the expression of AKT2 (Figure 3(a)), GLUT2 (Figure 3(b)), PK (Figure 3(c)), and PI3K (Figure 3(d)). Conversely, FAS expression was significantly higher in the MFB‐treated group (Figure 3(e)), while PEPCK expression did not show significant differences among treatments (Figure 3(f)).

Figure 3Expression of genes (∆∆Ct) related to glycolipid metabolism: (a) AKT2, (b) GLUT2, (c) PK, (d) PI3K, (e) FAS, and (f) PEPCK. An asterisk (^∗^) denotes a statistically significant difference relative to the control group; ^∗∗^ *p* < 0.01, ^∗∗∗^ *p* < 0.001, and ^∗∗∗∗^ *p* < 0.0001. Values are displayed as mean ± SD (*n = *3).

**Figure:**
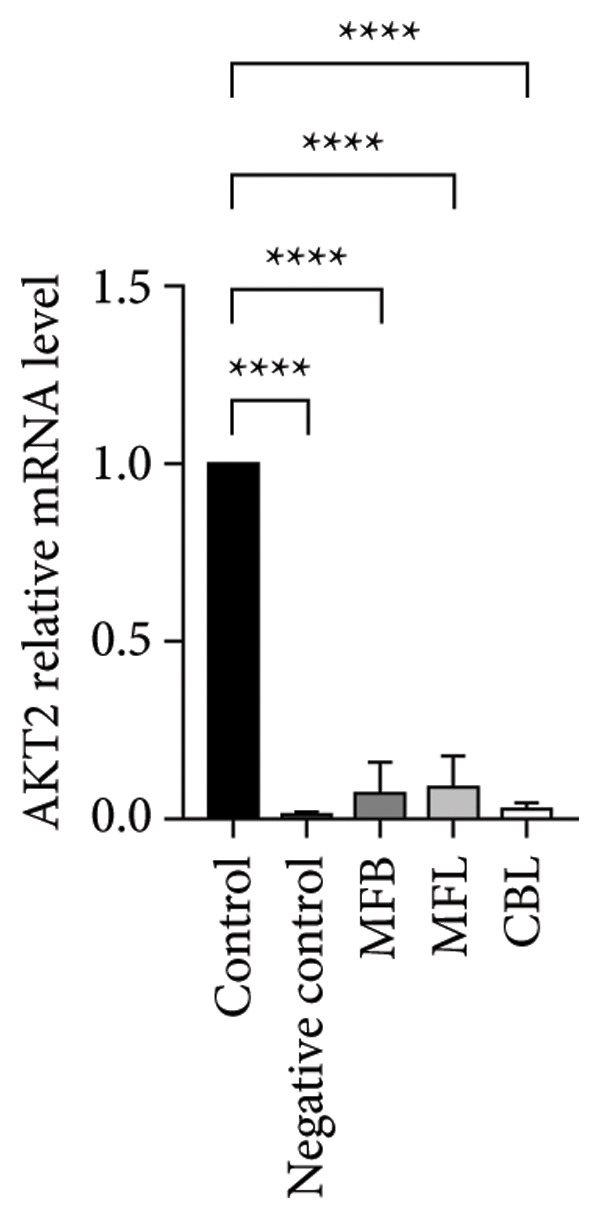
(a)

**Figure:**
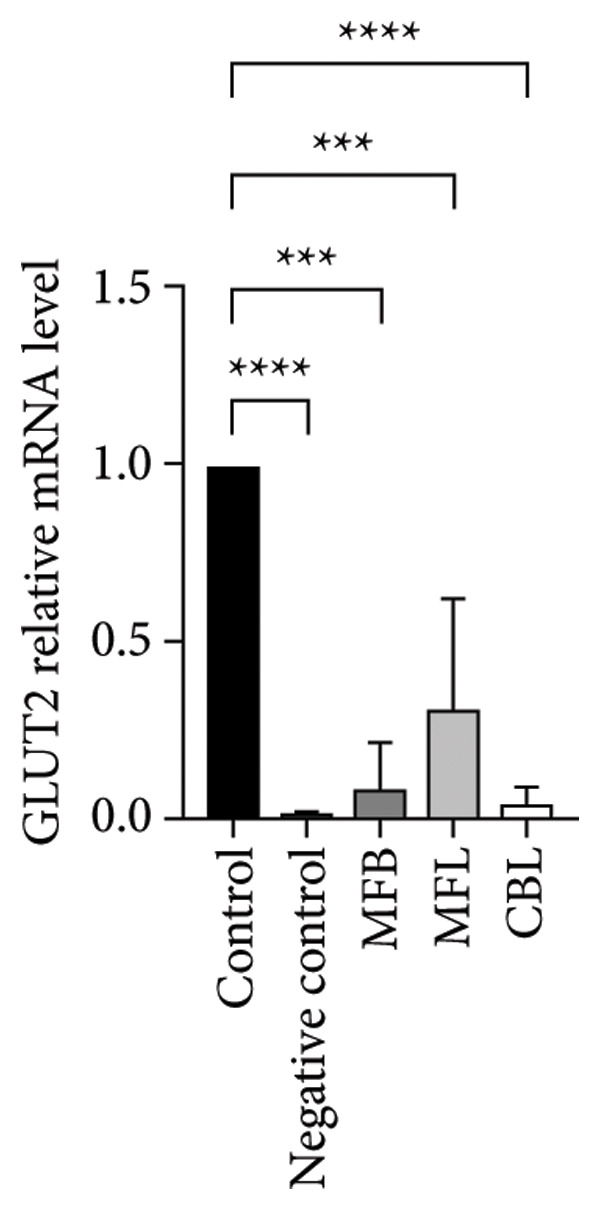
(b)

**Figure:**
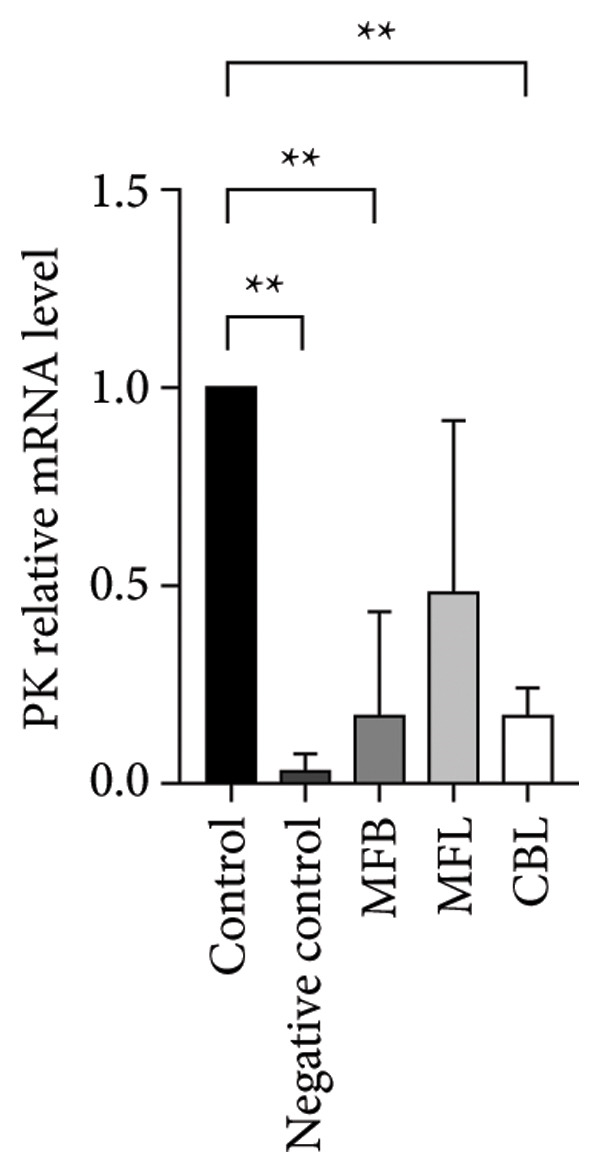
(c)

**Figure:**
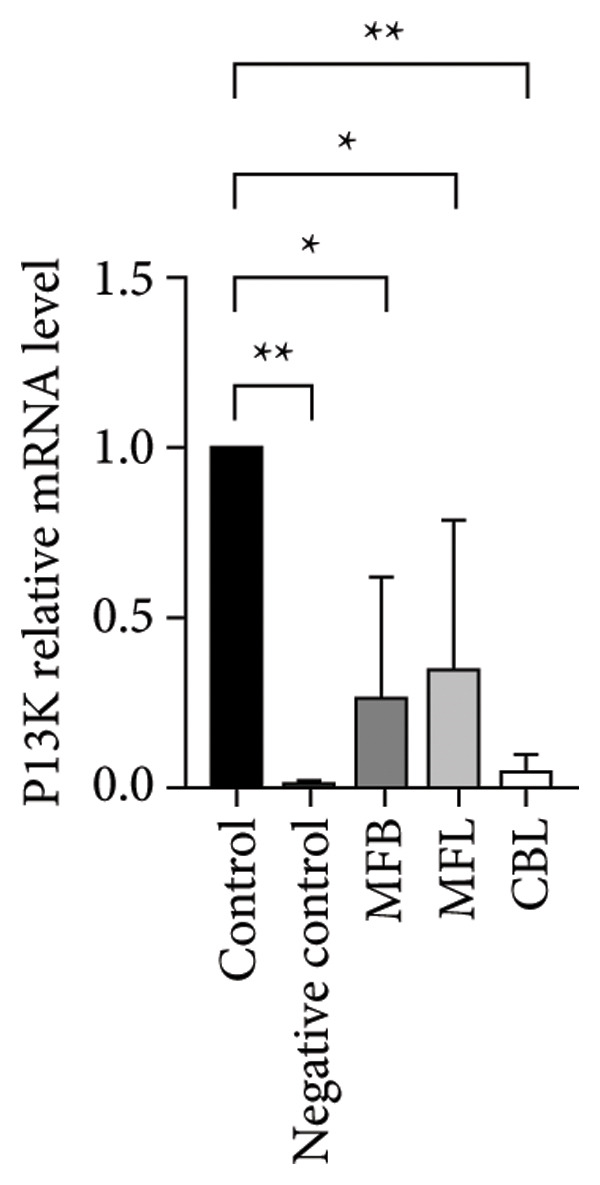
(d)

**Figure:**
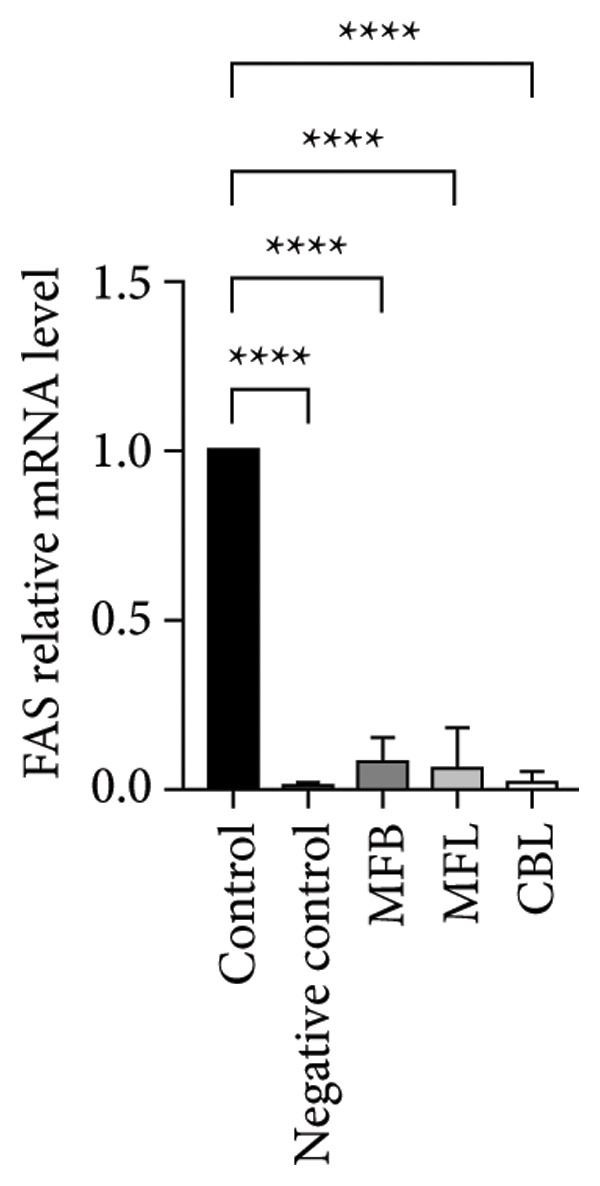
(e)

**Figure:**
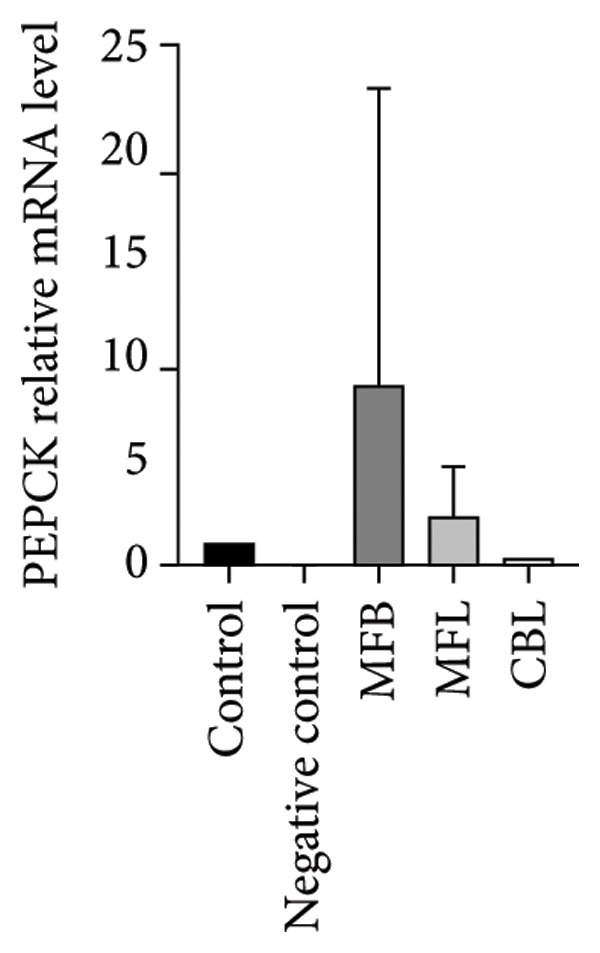
(f)

### 3.4. Plant Extract Improves Liver Structural Changes due to NPs

This study observed the percentage of normal hepatocytes, hepatocyte swelling, hepatocytes with necrosis, the number of Kupffer cells, the diameter of hepatic portal vein, and the diameter of central vein. As shown in Figure 4(a), the negative control had the lowest percentage of normal hepatocytes (64.86 ± 2.17%). The extract of CBL showed a significant difference compared to all groups (*p* < 0.05) and had the percentage of normal hepatocytes of 70.79 ± 0.8%. The same result was shown for the percentage of hepatocytes with necrosis. Figure 4(b) showed that administration of the plant extract in MFB, MFL, and CBL had significant differences compared to the negative control (*p* < 0.05).

Figure 4(a) Percentage of normal hepatocytes and (b) percentage of hepatocytes with necrosis. Values are represented as mean ± SD (*n* = 5). ^∗^ *p* < 0.05 and ^∗∗^ *p* < 0.01.

**Figure:**
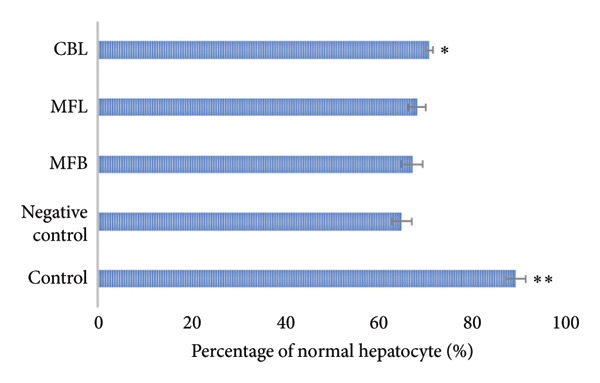
(a)

**Figure:**
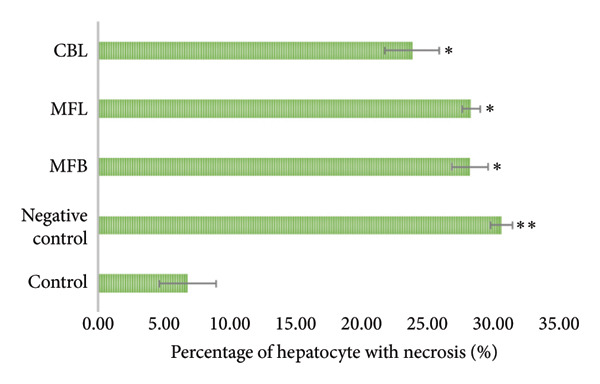
(b)

According to Figure 5(a), the percentage of hepatocyte swelling could be suppressed with the administration of plant extract. Administration of CBL significantly lowered the percentage of hepatocyte swelling compared to the negative control, MFB, and MFL (*p* < 0.05). The highest number of Kupffer cells was observed in the negative control (66.2 ± 2,13). Administration of the plant extract in all treatment groups could suppress the number of Kupffer cells significantly compared to the negative control (*p* < 0.05) (Figure 5(b)).

Figure 5(a) Percentage of hepatocytes swelling and (b) number of Kupffer cells. Values are represented as mean ± SD (*n* = 5). ^∗^ *p* < 0.05 and ^∗∗^ *p* < 0.01.

**Figure:**
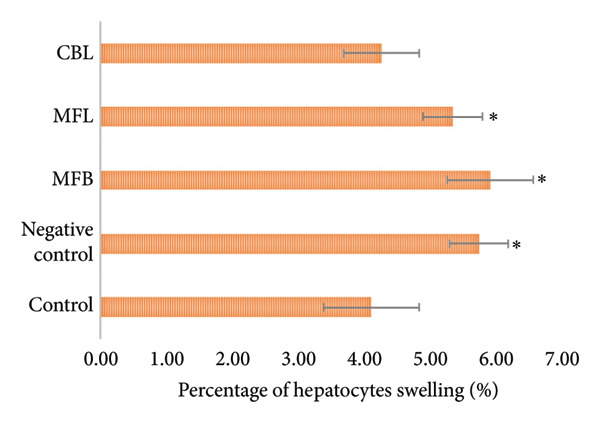
(a)

**Figure:**
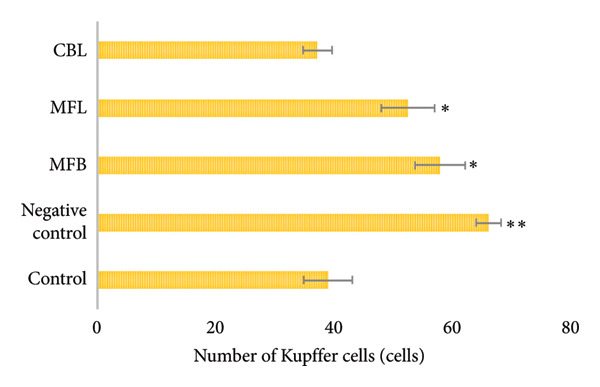
(b)

According to Figure 6, there was no significant difference in the diameter of the hepatic portal vein in all groups (*p* > 0.05). However, MFB, MFL, and CBL showed a significant difference in the diameter of the central vein compared to the negative control (*p* < 0.05).

Figure 6(a) The diameter of the hepatic portal vein and (b) the diameter of the central vein. Values are represented as mean ± SD (*n* = 5). ^∗^ *p* < 0.05 and ^∗∗^ *p* < 0.01.

**Figure:**
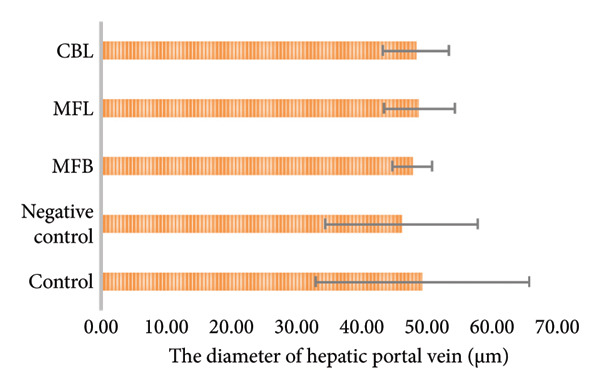
(a)

**Figure:**
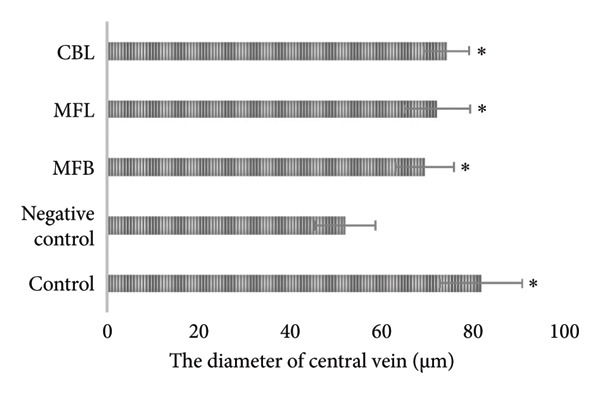
(b)

Administration of the NP in the negative control led to changes in hepatocytes and tissue structure compared to the control group, indicating toxic effects. Hepatocytes were arranged neatly, with large round nuclei, clear nucleoli, and peripheral chromatin distribution (Figure 7(a)). NP exposure caused changes in liver structure, with a large number of Kupffer cells observed in the sinusoid walls. Sinusoids experienced inflammation, indicating infiltration of erythrocytes and mononuclear cells around the sinusoids, hepatocyte degeneration, and necrosis (Figure 7(b)). Administration of the plant extract at 200 mg/kg showed recovery of hepatocyte structure similar to the control, with normal sinusoids containing many Kupffer cells, dominant normal hepatocytes, and decreasing necrosis (Figures 7(c), 7(d), and 7(e)).

Figure 7Rat liver section. (a) Control group showed normal liver structure and (b) rats exposed to NP (30 days) showed inflammation and necrosis; (c–e) rats exposed to NPs followed by plant extract (200 mg/kg MFB, MFL, and CBL) (HE staining, 400x). 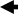: inflammation, 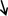: necrosis, and 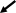: edema.

**Figure:**
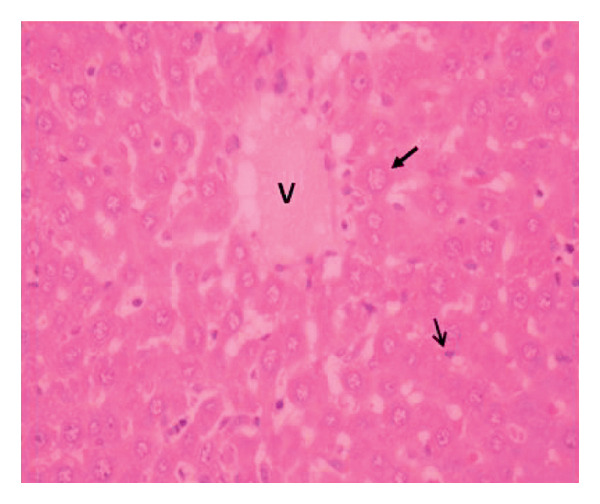
(a)

**Figure:**
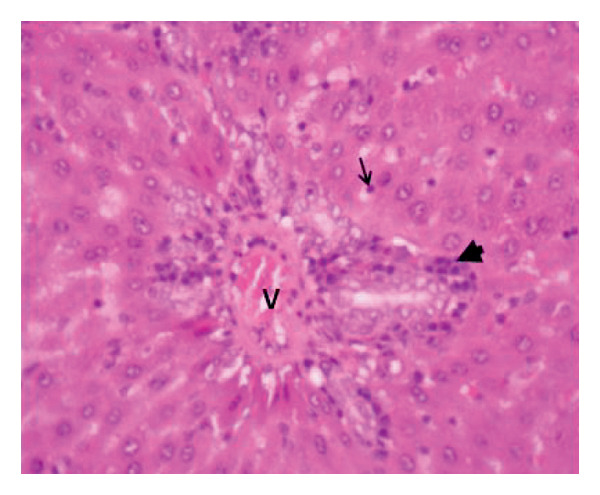
(b)

**Figure:**
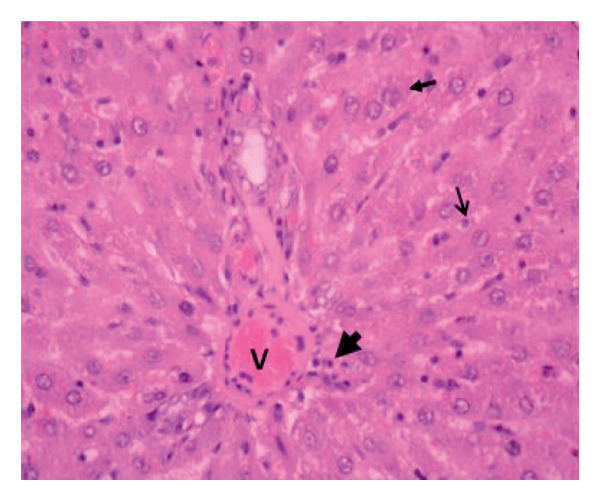
(c)

**Figure:**
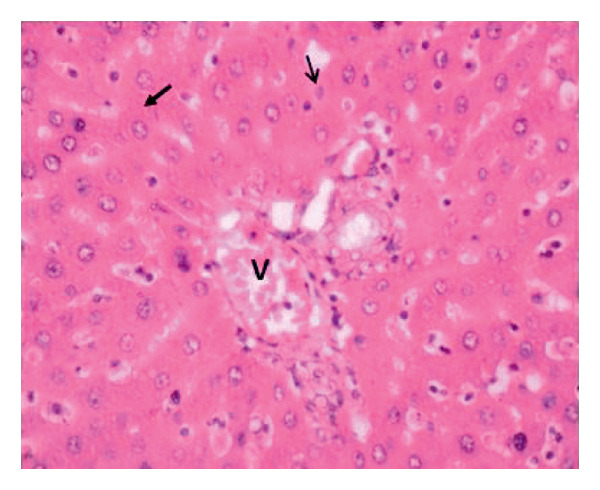
(d)

**Figure:**
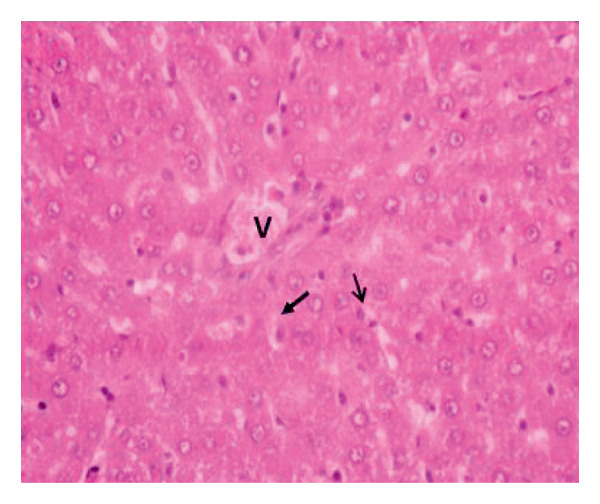
(e)

### 3.5. The Levels of SGOT, SGPT, Glucose, and ALP

In this study, SGOT and SGPT levels were observed as indicators of liver function after exposure to NP and plant extract treatment. The result showed that there were no significant differences between groups. However, there was an increasing level of SGOT in the group that was exposed to NP (32.2 ± 4.3 U/L) compared to the control group (31.4 ± 4.2 U/L). The addition of plant extract was able to decrease the SGOT levels compared to the group with NP exposure. Extract of CBL showed the lowest SGOT levels (28.7 ± 2.0 U/L) compared to the MFB (30.5 ± 3.4 U/L) and MFL (30.1 ± 0.8 U/L) (Figure 8(a)).

Figure 8(a) SGOT level of rats after NP exposure and plant extract administration, (b) SGPT level of rats after NP exposure and plant extract administration, (c) level of glucose of rats after NP exposure and plant extract administration, and (d) level of ALP of rats after NP exposure and plant extract administration. Values are represented as mean ± SD (*n* = 5). Different superscripts showed different significance.

**Figure:**
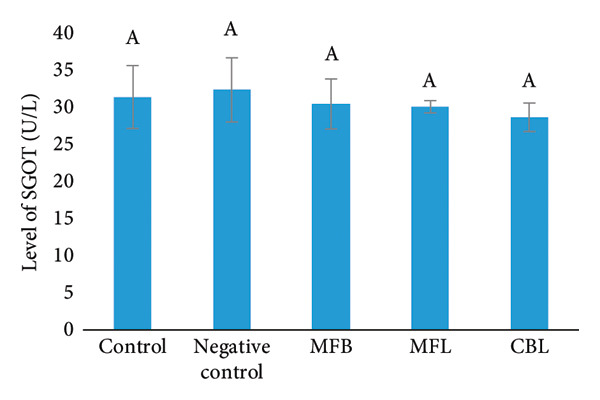
(a)

**Figure:**
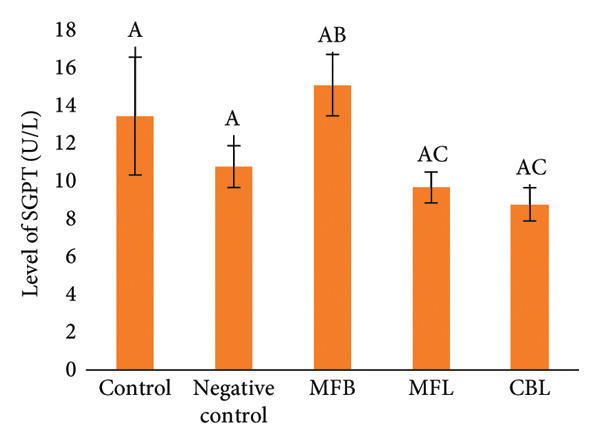
(b)

**Figure:**
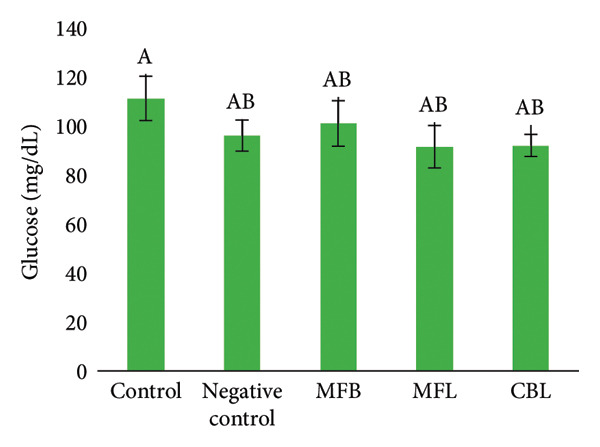
(c)

**Figure:**
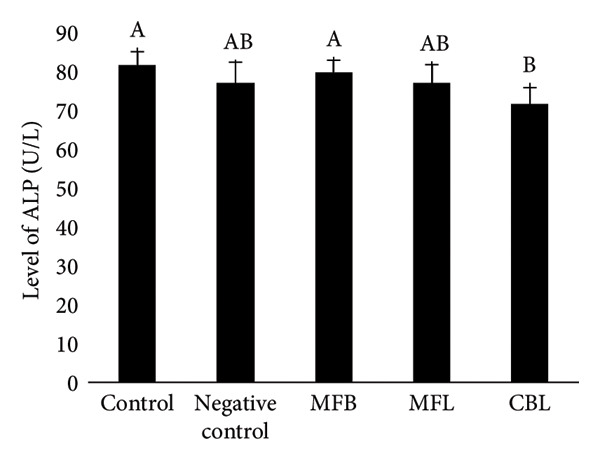
(d)

A different result was shown in the measurement of SGPT levels. The SGPT levels in the group that was exposed to NP decreased (10.8 ± 1.1 U/L) compared to the control group (13.46 ± 3.1 U/L). The SGPT levels in the MFB were decreased but did not show a significant difference (151 ± 16 U/L) compared to the NP exposed group (*p* < 005). The addition of MFL and CBL was able to decrease the levels of SGPT in the blood serum of rats compared to the negative group with the amount of (9.67 ± 0.8 U/L) and (8.8 ± 0.8 U/L), respectively (Figure 8(b)).

Glucose synthesis or glucogenesis occurs in the liver, and glucose levels can be an indicator of liver health. The exposure of NP decreased the glucose levels significantly (96 ± 6.35 mg/dL) compared to the control group (111.2 ± 9.03 mg/dL) (*p* < 0.05). The oral administration of the plant extract did not show significant differences in glucose levels compared to the negative group. Although there was no significant difference, there were increasing glucose levels in the MFB (101 ± 9.3 mg/dL). In the administration of MFL, the glucose levels were decreased (91.5 ± 8.6 mg/dL) compared to the negative group and there was a slight increase in glucose levels in the CBL group (92 ± 4.5 mg/dL) (Figure 8(c)).

Alkaline phosphatase (ALP) enzymes that are located in the liver act as protein converters into energy for cells. ALP will be released into the bloodstream during the stress condition, resulting in increased levels. However, in this study, there were no decreasing levels of ALP enzymes in the NP exposed group (77 ± 5.4 U/L) compared to the control group (81.72 ± 3.34 U/L) significantly (*p* < 0.05). The ALP enzyme levels in the MFB (79.85 ± 3.04 U/L) and MFL (77.1 ± 4.6 U/L) did not show a significant difference compared to the negative control (*p* < 0.05). The addition of CBL (71.56 ± 4.3 U/L) was able to reduce the glucose levels compared to the negative group (Figure 8(d)).

## 4. Discussion

The current state of environmental health is facing many pressures and presents significant challenges to human health. Pollution from plastic waste has become an urgent issue. Considerable information exists about NP pollution in our environment, including air pollution from vehicle emissions and waste incineration, contaminated water containing micro‐NP, sea salt derived from seawater evaporation, and cosmetics utilizing NP materials that impact health [62]. One of the effective solutions to address this issue is using a phytochemical extract. Phytochemical extracts possess unique advantages as they are natural, abundant, low‐residue, pollution‐free, and renewable sources. Their antioxidant properties can alleviate various forms of stress, including oxidative stress induced by NP [63].

According to confocal microscopy analysis, this study’s DAPI and Nile red staining indicated that NP entered the cell by endocytosis. Endocytosis enables the cell membrane to capture small compounds and transport them into the cytoplasm, nucleus, and nucleolus. A study conducted by Han et al. [64] stated that small‐sized polystyrene‐NP can enter cells by endocytosis and accumulate in the cytoplasm. Besides the cytoplasm, our study showed that NP can enter the nucleus and nucleolus of hepatocytes. The discovery of NP in the cytoplasm and nucleus indicates their ability to cross the cell membrane and nuclear membrane, potentially triggering oxidative stress and DNA damage. Staining with Nile red validated the presence of NP, which is consistent with previous reports on the bioaccumulation of NP in cellular tissues. This condition can lead to organelle dysfunction and affect apoptotic pathways.

Exposure to NP caused oxidative stress resulting in inflammation and apoptosis [65]. Apoptosis is a programmed cell death process that efficiently eliminates damaged cells [66]. This study showed increased levels of proapoptotic enzymes Caspase‐3, Caspase‐9, and Bax in the negative control with the administration of NP only. The Bax levels have risen due to the activation of the apoptotic cascade. This protein induces permeabilization of the outer mitochondrial membrane and promotes the release of Cytochrome C from the mitochondria into the cytoplasm.

This activity promotes the formation of proapoptotic structures. The intrinsic and extrinsic pathways are responsible for generating apoptotic signals [67]. Subsequently, Caspase‐9, the initiator of the intrinsic apoptosis pathway, becomes elevated. Activation of Caspase‐9 leads to the formation of Caspase‐3, which functions as an executor in the apoptosis process [68]. The apoptosis pathway is also influenced by the FAS cell surface death receptor. Activation of the FAS receptor, through binding with the FAS ligand, can increase the level of Caspase‐8, which is the initiator of the extrinsic apoptosis pathway [69]. Both intrinsic and extrinsic apoptosis pathways depend on Caspase‐3 to carry out apoptosis, leading to the cell nucleus disappearance.

Extract of MFB, MFL, and CBL could lower the Caspase‐3 level compared to the negative control. This result showed that the photochemical extract could inhibit apoptotic enzymes. Plant extract used in this study acts in the apoptosis extrinsic pathways because the plant extract lowered Caspase‐3 level, but the levels of Bax and Caspase‐9 were as high as in the negative control. The decrease in Caspase‐3 levels after the administration of plant extracts indicates the antiapoptotic potential of *C. burmannii* and *M. foetida* extracts. However, the lack of significant changes in Caspase‐9 and Bax may suggest that the protective mechanism mediated by the extracts is more related to the inhibition of the executioner apoptosis pathway (Caspase‐3) rather than the initiation pathway (Caspase‐9 and Bax). However, this study only measures certain elements of the apoptosis pathway, so it does not cover the entire mechanism of apoptosis.

Oxidative stress is known to be associated with the development of metabolic diseases including diabetes by damaging the structure and function of cellular proteins, impairing energy metabolism, altering cell signaling, and impairing cell transport mechanisms [70]. ROS including NP could impact glucose metabolism. Negative control with administration of NP only showed the lowest expression of all glycolipid metabolism–related genes such as PI3K, AKT, GLUT2, PEPCK, and PK among all the treatment groups. The activation of the PI3K gene occurs upon insulin binding to the insulin receptor. Subsequently, PI3K stimulates the activation of AKT, which plays a role in controlling glucose metabolism. GLUT2 facilitates the mobilization of glucose into the cell. Increased levels of PI3K, AKT, and GLUT2 indicate heightened activity in glucose uptake by the cell. The PEPCK signal is responsible for initiating gluconeogenesis from noncarbohydrate sources in the liver, converting oxaloacetate to phosphoenolpyruvate [71]. Meanwhile, PEP is responsible for converting phosphoenolpyruvate to pyruvate during glycolysis. All treatment groups administrated with plant extract (MFB, MFL, and CBL) exhibit the ability to regulate glycolipid metabolism by increasing the expression of PI3K, AKT, GLUT2, PEPCK, and PK genes. Plant extracts prevent the hyperglycemic state.

Our findings reveal changes in the expression of energy metabolism–related genes such as AKT2, PI3K, GLUT2, and PK after administration of plant extracts. The roles of AKT2 and PI3K in metabolic disorders are significant, as both are key components of the insulin signaling pathway. Activation of PI3K induces phosphorylation of AKT2, which plays a crucial role in enhancing glucose uptake via GLUT2 and GLUT4 transporters. NP exposure that disrupts this pathway can decrease insulin sensitivity, thus increasing the risk of insulin resistance, a hallmark of Type 2 diabetes. Our finding that the expression of AKT2 and PI3K remains disrupted after NP exposure supports this hypothesis. The increase in AKT2 and GLUT2 after the administration of *Mangifera foetida* extract suggests potential improvement in the insulin signaling pathway, which could serve as a relevant mitigation strategy. The increased expression of AKT2, GLUT2, and PK genes after administration of *M. foetida* extract indicates a positive effect on energy metabolism pathways, which may support cellular regeneration. The AKT pathway plays a role in promoting cell survival, while the increased GLUT2 expression suggests an improvement in glucose uptake mechanisms. In the long term, NP exposure can cause systemic inflammation and chronic oxidative stress, contributing to metabolic dysfunction. If left unaddressed, this condition may exacerbate the risk of metabolic syndrome, including Type 2 diabetes, cardiovascular disease, and nonalcoholic fatty liver disease (NAFLD).

Serving as a major metabolic organ, the liver regulates various metabolic pathways that connect different tissues and organs. It serves as the location for gluconeogenesis [72]. Glucose in the form of glycogen is a source of blood glucose [73]. The liver also undergoes the process of protein oxidation, providing energy. The result of protein metabolism forms amino acids, subsequently broken down into keto acids and ammonia [74]. Furthermore, the liver serves as a principal site for the metabolism of harmful chemicals. The liver’s vital role involves detoxifying blood by processing various waste products in hemoglobin [75]. The liver synthesizes and secretes several types of enzymes for optimal regulation, including SGOT, SGPT, as well as ALP. Exposure to NP is suspected to trigger alterations in liver metabolism pathways, key metabolic enzymes, and enzymes stimulated by oxidative stress [76]. This study showed that the administration of NP could elevate the level of SGOT. Administration of the plant extract in MFB, MFL, and CBL showed a slight decrease in the level of SGOT and SGPT.

In the observation of liver histology, there is a change in liver structure due to NP exposure in the negative control. Structural changes show hepatocytes undergoing necrosis, swelling, addition of Kupffer cells, and decrease of central vein diameter. In vivo administration of the plant extract caused hepatoprotective activity against NP. This protective mechanism may be related to the extract’s ability to reduce oxidative stress, inhibit inflammation, and support cellular regeneration. In line with the study conducted by Abbas et al. and Fajri et al. [49, 50], at a dose of 200 mg/kg, CBL and MFL extracts did not show significant changes in liver biochemical parameters and did not cause histological damage to the organ. This indicates that this dose is safe over 30 days in the rat model.

In other studies, CBL exhibited antibacterial, anti‐inflammatory, and antioxidant activity [77]. CBL containing flavonoids, saponins, tannins, polyphenols, quinones, triterpenoids, and cinnamaldehyde acts as an antioxidant [78]. These compounds contribute to the body’s protection from oxidative damage caused by free radicals. Flavonoids have been clinically proven to prevent diabetes, cardiovascular disorders, and kidney abnormalities based on their antioxidant potential [79]. The presence of bioactive compounds such as flavonoids can mitigate oxidative stress and support cellular signaling pathways such as AKT. *M. foetida* extract, which increases AKT2 and GLUT2, is more prominent in supporting energy metabolism, while *C. burmannii* has a stronger effect in protecting hepatocytes from necrosis.

The decrease in intracellular NP observed in the treated groups may be explained by several mechanisms related to the bioactive compounds present in *C. burmannii* and *M. foetida*. These extracts are rich in flavonoids and polyphenols, which are known to stabilize cell membranes and reduce permeability, thereby limiting passive NP entry. Moreover, flavonoids have been shown to modulate endocytosis pathways and enhance autophagic activity, potentially promoting the degradation or expulsion of internalized NP via lysosomal clearance. Antioxidant and anti‐inflammatory activities of the extracts may also reduce oxidative stress–mediated cellular uptake of foreign particles, indirectly lowering NP accumulation. These mechanisms may work synergistically to reduce NP retention in cells and mitigate associated toxicity.

However, it is possible that not all bioactive compounds were successfully isolated, so the observed effects may be attributed to a mixture of compounds. For future studies, it is essential to evaluate additional biomarkers beyond Caspase‐3, Caspase‐9, and Bax to comprehensively delineate the apoptosis pathway. Furthermore, other liver function indicators should be incorporated, as SGOT, SGPT, ALP, and glucose are broad markers and may lack the sensitivity required to detect NP‐induced disruptions. Subtle alterations in hepatic function may remain undetected with these parameters.

## 5. Conclusion

This study confirms that NP could increase apoptosis protein activity and disrupt hepatocyte structure. Plant extracts (MFB, MFL, and CBL) could be effective materials that exhibit exogenous antioxidant activity against NP. These extracts play a critical role in enhancing cell survival by directly inhibiting proapoptotic signals and regulating glycolipid metabolism. Given their established antioxidant and hepatoprotective properties, these extracts have the potential to be developed as preventive or therapeutic interventions such as dietary supplements and nutraceutical products to mitigate the increasing concern surrounding NP exposure in both environmental and clinical contexts.

## Conflicts of Interest

The authors declare no conflicts of interest.

## Funding

This research was funded by Universitas Airlangga, Indonesia, fiscal year 2023, research funding batch 2 number: 1711/UN3.LPPM/PT.01.03/2023.

## Data Availability

The authors confirm that the data supporting the findings of this study are available within the article.
